# The Improvement of Skin Whitening of Phenylethyl Resorcinol by Nanostructured Lipid Carriers

**DOI:** 10.3390/nano7090241

**Published:** 2017-08-28

**Authors:** Bo-Sik Kim, Young-Guk Na, Jae-Hwan Choi, Inhye Kim, Eunji Lee, Sung-Yeon Kim, Jae-Young Lee, Cheong-Weon Cho

**Affiliations:** 1 College of Pharmacy and Institute of Drug Research and Development, Chungnam National University, 99 Daehak-ro, Yuseong-gu, Daejeon 34134, South Korea; kimbosik1098@naver.com (B.-S.K.); youngguk@cnu.ac.kr (Y.-G.N.); jaeyoung@cnu.ac.kr (J.-Y.L.); 2 IN2BIO Research and Development center, Suwon 16681, South Korea; shevlove@hanmail.net; 3 Graduate School of Analytical Science and Technology, Chungnam National University, 99 Daehak-ro, Yuseong-gu, Daejeon 34134, South Korea; inhyekim@cnu.ac.kr (I.K.); eunjilee@cnu.ac.kr (E.L.); 4 Institute of Pharmaceutical Research and Development, College of Pharmacy, Wonkwang University, Iksan 54538, South Korea; sungykim@wku.ac.kr

**Keywords:** nanostructured lipid carriers, phenylethyl resorcinol, topical drug delivery, stability, cellular tyrosinase inhibition assay

## Abstract

Phenylethyl resorcinol (4-(1-phenylethyl)1,3-benzenediol) (PR) is a new whitening agent that has been found to have the ability to inhibit tyrosinase activity. However, the application of PR is limited by photo instability and poor solubility. PR-loaded nanostructured lipid carriers (PR-NLCs) were prepared by the hot-melted ultrasonic method. Glycerol monostearate and olive oil were selected as the solid lipid and liquid lipid for considering the solubility of PR in liquid lipid and partition coefficient of PR in solid lipid, respectively. The particle size and polydispersity index of PR-NLCs were 57.9 ± 1.3 nm and 0.24 ± 0.01, respectively. The encapsulation efficiency and loading capacity of PR-NLCs were 93.1 ± 4.2% and 8.5 ± 0.4%, respectively. The stability test demonstrated that the incorporation of PR into NLCs conferred excellent physicochemical stability and photo stability for at least three months at 4 °C in the dark and 25 °C under daylight. In vitro release of PR-NLCs revealed a sustained release pattern. Cellular tyrosinase assay showed that PR-NLCs could significantly inhibit tyrosinase activity in melanoma cells, suggesting that NLCs can be used as a biocompatible nanocarrier for the effective delivery of skin whitening agents.

## 1. Introduction

**Figure 1:**
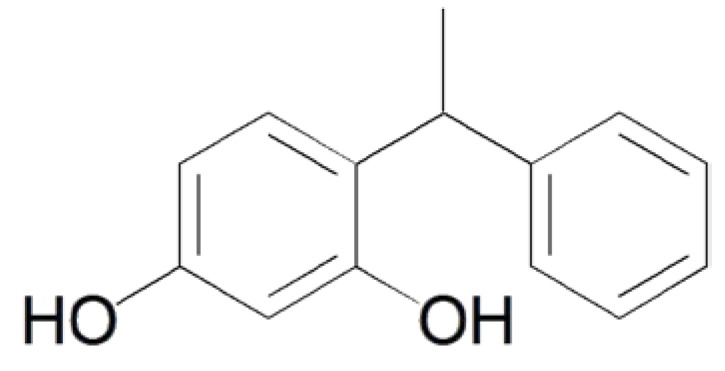
Structure of 4-(1-phenylethyl)1,3-benzenediol (PR).

Phenylethyl resorcinol (4-(1-phenylethyl)1,3-benzenediol) (PR) (Figure 1) is a new whitening agent that has been found to have the ability to inhibit tyrosinase activity [1]. PR has been shown to serve as a whitening and brightening ingredient in skin care products, hair lightening products, and cosmetics. It is a synthetic compound that is partially derived from natural lightening compounds found in Scotch pine bark. According to research, it is one of the highest tyrosinase inhibitors, 22 times more potent than kojic acid [2]. When directly compared to β-arbutin, PR was shown to be over 100 times as effective at lightening hair. Also, it served as an antioxidant agent—better than vitamin E, vitamin C, and butylated hydroxytoluene. However, the application problems of PR were due to its light instability and poor water solubility. The poor water solubility may limit its absorption, while its photo instability may render the topically applied PR ineffective [3]. Therefore, there is a need for appropriate delivery vehicles that can improve the photo stability and water solubility of PR.

In recent years, nanotechnology has been intensively studied in many fields such as computers, engineering, and electronics, as well as pharmaceutical technology. In the pharmaceutical field, drug delivery systems with a nanosize range have shown increased solubility, enhanced dissolution rate, and improved bioavailability [4]. Nanoparticles can be prepared using different kinds of materials—for example, biodegradable and biocompatible polymers, phospholipids, surfactants, and lipids [5]. Nanoparticles prepared from lipid materials have been demonstrated to have the advantages of biocompatibility, biodegradability, drug targeting, modified release, lack of organic solvent during the production process, and ease of large-scale production [6].

NLCs with more imperfections in the crystal structure as compared to solid lipid nanoparticles (SLNs) are prepared using a blend of solid lipid and spatially different liquid lipids. These imperfections contribute to improved drug loading and reduced drug expulsion during storage [7,8]. Therefore, NLCs that are lipid nanoparticles or colloidal carriers have been explored as potential topical delivery vehicles. NLCs have been reported to offer several advantages over conventional topical products owing to their ability to prolong drug release, mitigate skin irritation, and protect the drug from potential degrading opportunities. Additionally, the high specific surface area of the particles ensures excellent contact with the affected site on the skin, facilitating the more efficient transfer of the drug [9]. 

Recently, allergic contact dermatitis caused by PR has been reported [10]. Therefore, we need to determine the concentration that causes dermatitis in order to effectively apply PR. Thus, PR formulated with NLCs can be used at lower concentrations because NLCs have the ability to increase solubility and absorption.

We prepared NLCs through screening of PR in lipids and evaluated PR-NLCs by in vitro release, cytotoxicity, cellular uptake study, and a cellular tyrosinase inhibition assay. 

## 2. Results and Discussion

### 2.1. Screening of PR in Solid and Liquid Lipids

**Figure 2:**
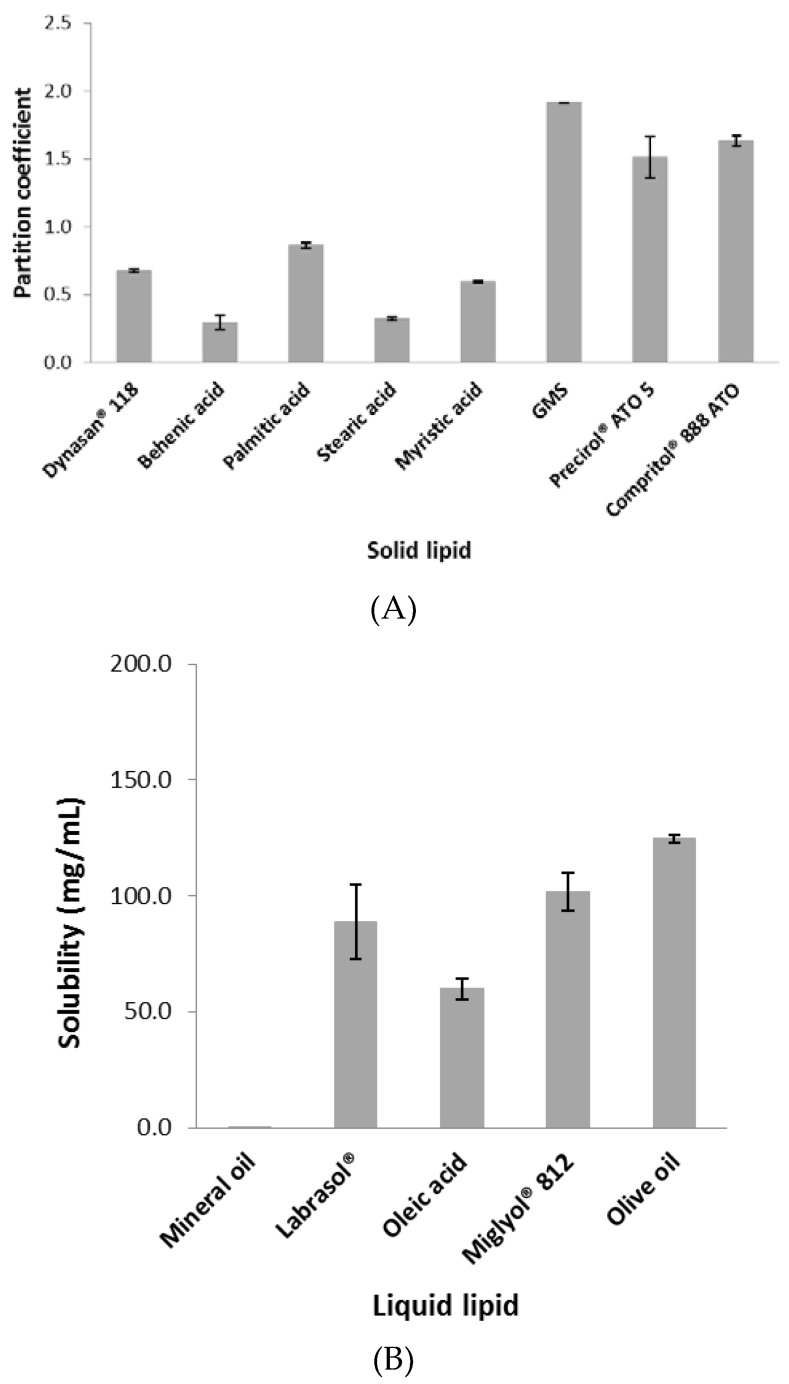
(**A**) The partition coefficient of PR in solid lipids (*n* = 3, mean ± standard deviation (SD)); (**B**) the solubility of PR in liquid lipids (*n* = 3, mean ± SD).

The partition coefficient and solubility of PR in the lipid matrix are major factors determining encapsulation efficiency (EE) and loading capacity (LC) in the NLCs [11]. Therefore, we investigated the partition coefficient of PR in solid lipids and the solubility of PR in liquid lipids to determine conditions for loading the maximum amount of PR into the NLC. For solid lipids, the partition coefficient of PR in Dynasan^®^ 118, behenic acid, palmitic acid, stearic acid, myristic acid, glycerol monostearate (GMS), Precirol^®^ ATO 5, or Compritol^®^ 888 ATO is shown in Figure 2A. GMS showed the highest partition coefficient value of PR. For liquid lipids, the solubility of PR in mineral oil, Labrasol^®^, oleic acid, Miglyol^®^ 812, or olive oil is seen in Figure 2B. Olive oil showed the highest solubility of PR. Based on these results, GMS and olive oil were selected for the preparation of NLCs. There has been a report of miconazole nitrate-loaded NLCs prepared using GMS and olive oil [12].

### 2.2. Measurements of Particle Size and Polydispersity Index (PDI)

The particle size and PDI are the physical properties of the colloidal dispersion determining stability of the formulation [13]. As shown in Table 1, the particle sizes of blank-NLCs and PR-NLCs were found to be 55.6 ± 1.2 nm and 57.9 ± 1.3 nm, respectively. The increase of particle size of PR-NLCs as compared to that of blank-NLCs could be due to the loading of PR in the lipid matrix. For both the formulations, PDI was below 0.25, which indicated the narrow size distribution of the nanoparticles [14].

### 2.3. Determination of EE and LC

The EE and LC are important factors for preparation technology [15]. The EE and LC of PR in PR-NLCs were measured using an ultrafiltration method. The result showed that the permeation rate was 100.1 ± 0.2%, indicating that the ultrafiltration membrane had no accumulation to the PR and the method was accurate. The EE and LC of PR-NLCs were 93.1 ± 4.2% and 8.5 ± 0.4%, respectively. It was found that the addition of a liquid lipid to a solid lipid leads to an increase in EE and LC because of minimizing the leak of PR from lipids [13].

### 2.4. Transmission Electron Microscopy (TEM) Analysis

**Figure 3:**
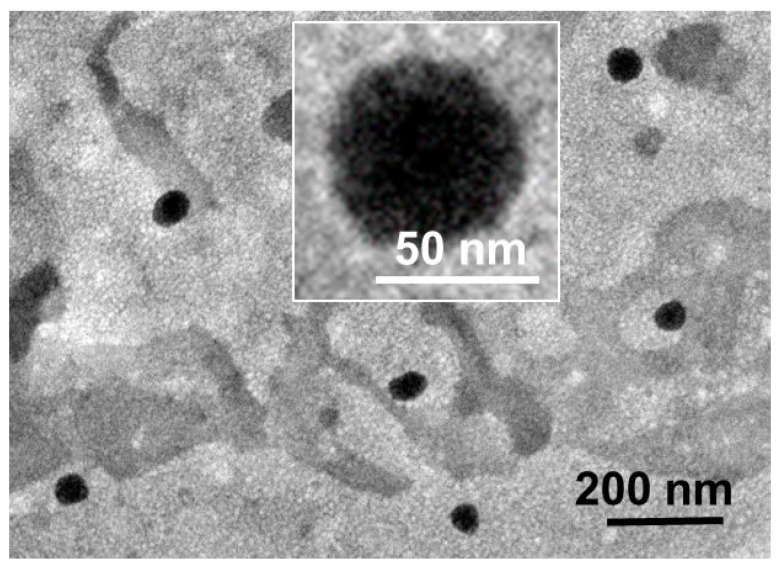
TEM image of PR-NLCs (scale bar 200.0 nm).

TEM images determined that PR-NLCs are of spherical morphology and did not adhere to each other (Figure 3). A gloom-like shadow could be seen around the particulate, possibly due to unevenly dyeing the background with uranyl acetate. The staining agent, uranyl acetate, binds to the carboxylic group of the surfactant; the density of the carboxylic group is more intense in a lipid as compared to a surfactant, showing that the lipid was dispersed with the vesicle type [16]. From the TEM analysis, the particle size of the PR-NLCs was in good agreement with the results obtained by dynamic light scattering (DLS).

### 2.5. Fourier-Transform Infrared (FT-IR) Analysis

**Figure 4:**
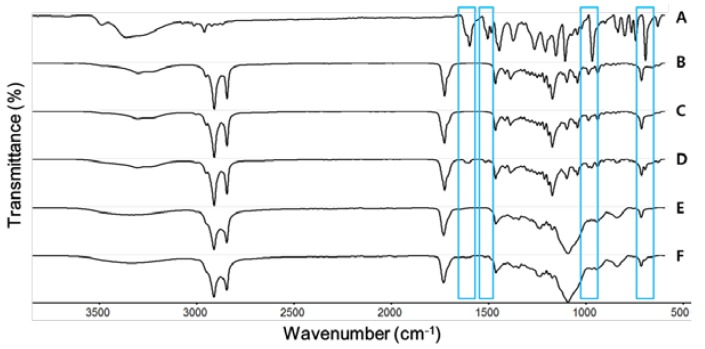
FT-IR spectra of (**A**) PR only; (**B**) GMS; (**C**) lipid melt; (**D**) PR-lipid melt; (**E**) freeze-dried blank-NLCs; (**F**) freeze-dried PR-NLCs.

FT-IR spectroscopic studies were performed to find out the possible interaction and complex formation between PR and lipid during the preparation of the NLCs. The FT-IR spectrum of the PR only revealed absorption bands at 1601, 1508, 971, and 697 cm^−1^ (Figure 4). These were the characteristic peaks of PR and these characteristic peaks were only present in the PR–lipid melt. FT-IR spectra of freeze-dried blank-NLCs and freeze-dried PR-NLCs were almost the same. This indicated the absence of any physical or chemical interaction between PR and compositions of NLCs. In the FT-IR spectrum of freeze-dried PR-NLCs, peaks corresponding to PR disappeared or were buried in the peaks of freeze-dried blank-NLCs, indicating PR entrapment in lipid matrix. This could be explained by the drug-enriched core model for the incorporation of active compounds into NLCs [3].

### 2.6. Differential Scanning Calorimetry (DSC) Analysis

**Figure 5:**
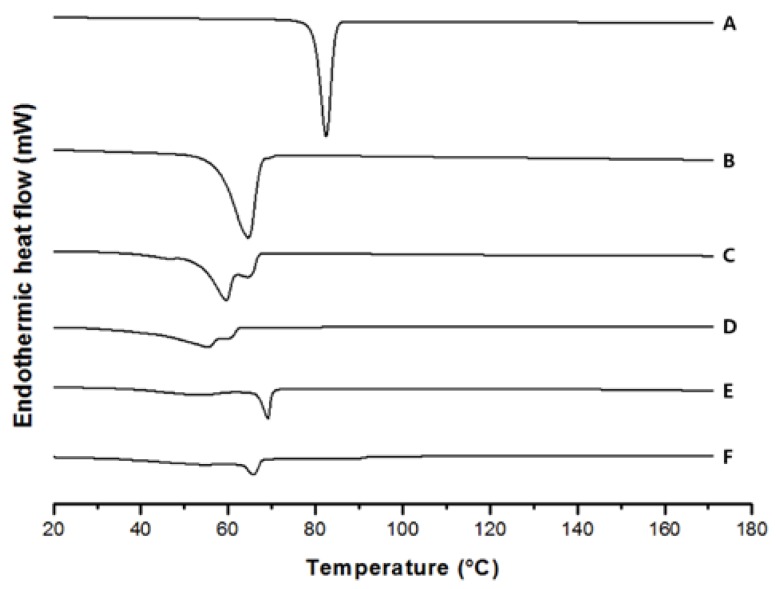
DSC thermograms of (**A**) PR only; (**B**) GMS; (**C**) lipid melt; (**D**) PR-lipid melt; (**E**) freeze-dried blank-NLCs; (**F**) freeze-dried PR-NLCs.

In order to investigate the changes of thermal characteristics, DSC experiments were performed. As shown in Figure 5, the sharp endothermic peak of PR observed at 81.88 °C, and the endothermic peak of GMS used as a solid lipid was observed at 64.36 °C. For blank-NLCs and PR-NLCs, endothermic peaks were observed at 68.86 and 65.59 °C, respectively. The thermogram of PR-NLCs did not show an endothermic peak for PR. This suggests that PR was not in a crystalline state but in an amorphous state or molecularly dispersed structure of the drug in a lipid matrix [17,18]. Also, the endothermic peak of PR-NLCs was shifted to a lower temperature compared to that of blank-NLCs. Incorporation of the drug inside the lipid matrix results in an increase in the number of defects in the lipid crystal lattice, and hence causes a decrease in the melting point of the lipid in the NLC formulations [19,20].

### 2.7. X-Ray Diffraction (XRD) Analysis

**Figure 6:**
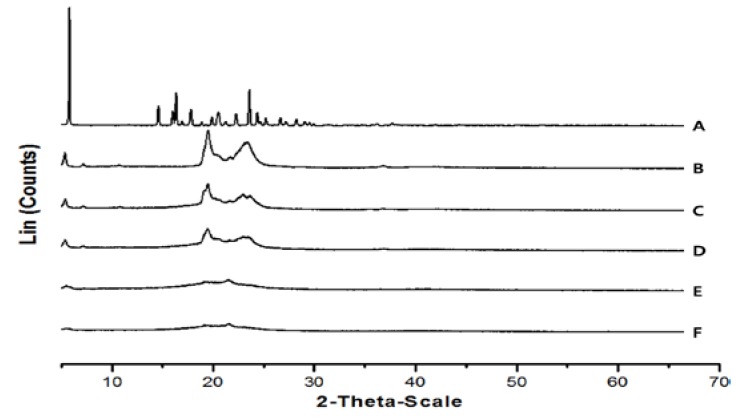
XRD patterns of (**A**) PR only; (**B**) GMS; (**C**) lipid melt; (**D**) PR–lipid melt; (**E**) freeze-dried blank-NLCs; (**F**) freeze-dried PR-NLCs.

The XRD pattern for PR only revealed principal peaks characteristic of its crystal form (Figure 6). The XRD patterns of GMS, lipid melt, and PR-lipid melt were almost the same. The principal peaks of PR were absent in PR-lipid melt and freeze-dried PR-NLCs. Furthermore, the principal peak of freeze-dried blank-NLCs did not shift in freeze-dried PR-NLCs but had a reduced intensity. These changes can be attributed to the incorporation of PR between parts of the crystal lattice of the lipid, leading to a change in the crystallinity of PR-NLCs. Thus, these results in the diffraction pattern support a conversion from the crystalline drug to an amorphous form and indicate PR entrapment in the lipid matrix [21,22].

### 2.8. Stability Test

**Figure 7:**
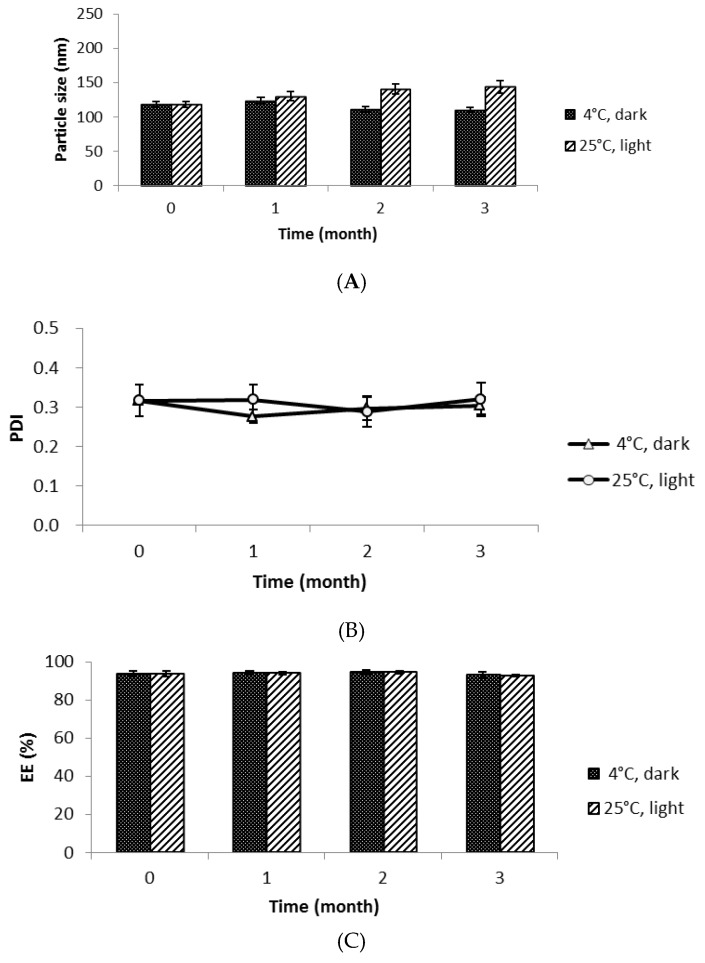
The stability of freeze-dried PR-NLCs over the course of three months. (**A**) The particle size; (**B**) PDI; (**C**) EE (*n* = 3, mean ± SD).

Freeze-drying is a good way to store NLCs for stability over a long period of time. The crystallization of ice might damage the surfactant film around the nanoparticle due to a freezing-out effect and also cause particle aggregation during the resolubilization or redispersion process [23]. The cryoprotectant forms a glassy/vitreous coating around the nanoparticles, protecting them against stresses like mechanical stress of ice crystals and thereby preventing aggregation [24]. Trehalose is a disaccharide formed of an α,α-1,1-glucoside bond between two α-glucose units. It is used as a cryoprotectant for NLCs because of its ability to preserve their original size and structure after freeze-drying [25]. For freeze-dried PR-NLCs, the particle size doubled and PDI displayed a slight increase up to 0.3 compared to non-freeze-dried PR-NLCs. However, the particle size and PDI of freeze-dried PR-NLCs was still in the acceptable range [26]. When PR-NLCs were stored in the solution state, they were precipitated. So, we stored PR-NLCs in the solid state and measured the content and particle size after reconstitution in distilled water. The stability of freeze-dried PR-NLCs was evaluated by measuring the particle size, PDI, and EE at 4 °C in the dark and 25 °C in the daylight during a three-month storage. As shown in Figure 7, the PDI and EE of freeze-dried PR-NLCs did not change over three months. Concerning the freeze-dried PR-NLCs stored at 25 °C in daylight, with increasing storage time, the particle size increased 25 nm over its initial size. However, the particle size below 200 nm after three months was still acceptable. The results of this study suggest that NLCs could be a way to enhance the stability of the formulation. Also, these data demonstrated that the incorporation of PR into NLCs could lend excellent physicochemical stability and photo stability for at least three months at 4 °C in the dark and 25 °C in the daylight.

### 2.9. In Vitro Release Study

**Figure 8:**
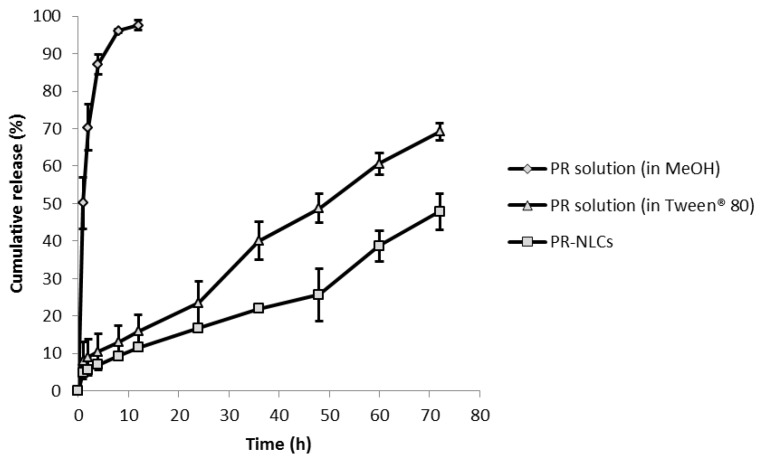
In vitro release profiles of PR solution and PR-NLCs (*n* = 3, mean ± SD).

For PR-solution in Tween^®^ 80 and PR-NLCs, the PR showed a release of 69% and 48% at 72 h, respectively (Figure 8). The cumulative release of PR from PR-NLCs was slower than that of the PR solution in Tween^®^ 80. A PR solution in methanol showed a fast release pattern. PR-NLCs have exhibited a sustained release pattern without bursts. This phenomenon is attributed to the formation of a drug-enriched core model that will slow down release of the drug [27].

### 2.10. Cytotoxicity Study

**Figure 9:**
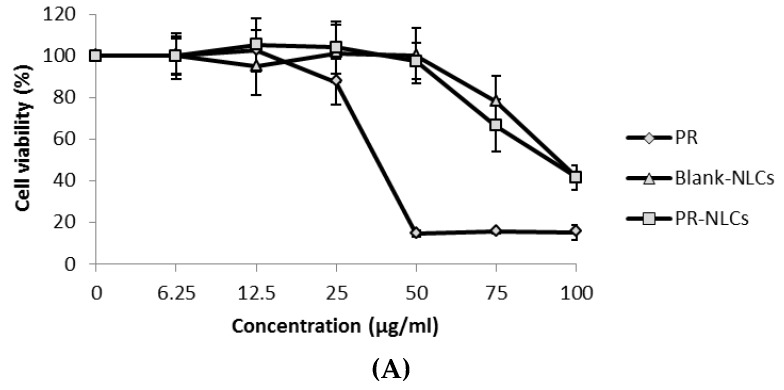

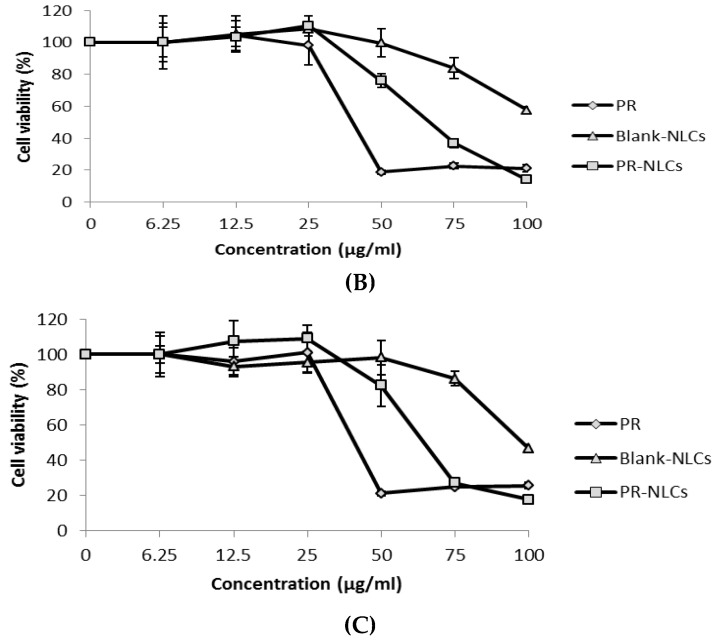
In vitro cytotoxicity of PR solution, blank-NLCs, and PR-NLCs in HaCaT cells for (**A**) 24 h; (**B**) 48 h; (**C**) 72 h (*n* = 3, mean ± SD).

To determine the potential cytotoxic activity of PR-NLCs, the cell viability was evaluated for HaCaT cells. Figure 9 shows the cell viability of HaCaT cells treated with PR solution, blank-NLCs, and PR-NLCs for 24, 48, and 72 h. The cell viability of each sample was observed for different PR concentrations. In all cases, with increasing concentration of PR, cell viability decreased. In this study, the cell viability of blank-NLCs was evaluated with a lipid concentration equivalent to that of PR-NLCs. Usually, cell viability >70% is considered “no toxicity” [28]. Meanwhile, cell viability <50% is considered “irritation” [29]. The results of the cell viability suggest that the blank-NLCs were nontoxic even at a concentration of 75 μg/mL. Also, it is obvious that the cell viability of PR-NLCs on HaCaT cells was different to that of the PR solution at the different time points. The PR solution showed cytotoxicity at a concentration of 50 μg/mL. On the other hand, PR-NLCs exhibited more than 70% cell viability at a concentration of 50 μg/mL. This might be explained by the slow release of PR from PR-NLCs. Unlike the occurrence of concentration spikes that appeared during the treatment with free PR, PR incorporated into the lipid matrix slowly diffuses and releases to the outer environment, thereby minimizing the cytotoxic effect [30]. It was found that the cell viability was related to the release study. 

### 2.11. Cellular Uptake Study

**Figure 10:**
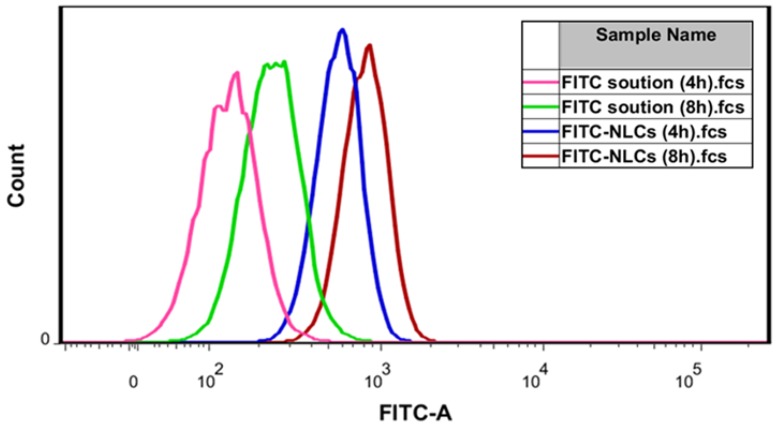
FITC intensity in B16F10 cells treated with solution or NLCs.

Figure 10 shows how the intensity of cells became stronger depending on formulation and time, which indicates that the level of cellular uptake was increased. The mean fluorescent intensity in melanoma cells treated with fluorescein isothiocyanate-NLCs (FITC-NLCs) was 580 mean fluorescence intensity (MFI) and 836 MFI after 4 and 8 h incubation, respectively, while it was 138 and 240 MFI in melanoma cells treated with FITC solution after 4 and 8 h incubation, respectively. The fluorescent intensity in melanoma cells treated with FITC-NLCs was 4.2-fold and 3.4-fold higher than that of FITC solution at 4 and 8 h incubation, respectively. Furthermore, the FITC intensity of each formulation increased according to the increased incubation time. After an 8-h incubation with FITC solution, the fluorescence intensity was 1.8-fold higher than after a 4-h incubation. After an 8-h incubation with FITC-NLCs, the fluorescence intensity was 1.4-fold higher than after a 4-h incubation.

**Figure 11:**
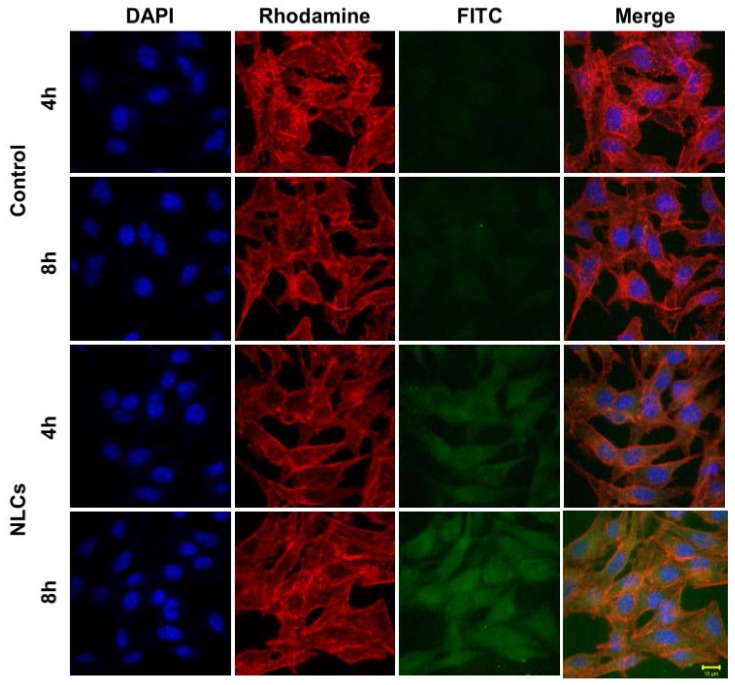
Fluorescent microscopic images of FITC solution and FITC-NLCs. Blue is the cell nuclei, red is the cell cytoplasm, and green is FITC. The diameter of the nucleus is 10 μm.

To evaluate whether the prepared NLCs could efficiently deliver PR into melanoma cells, cellular uptake of NLCs was also observed by confocal laser scanning microscope (Figure 11). FITC-NLCs mostly accumulated in the cell membrane region. These results indicate that the cellular uptake or accumulation of FITC-NLCs in melanoma cells was enhanced as compared to that of a FITC solution. According to the literature, nanoparticle structure and surface properties are crucial in dictating cellular fate [31]. The literature also indicates that NLCs were taken up in a size-dependent manner and clathrin-mediated endocytosis was proposed to be the major pathway for the uptake of particles with a size below 200 nm [32]. Moreover, the components of the lipid and lecithin in surfactants add affinity to NLCs to the cell membranes, supporting the involvement of adsorptive endocytosis, which requires higher affinities than those taken up by receptor-mediated endocytosis [33]. Therefore, these data indicate that NLCs could be efficiently taken up into melanoma cells as a whitening agent.

### 2.12. Cellular Tyrosinase Inhibition Study

**Figure 12:**
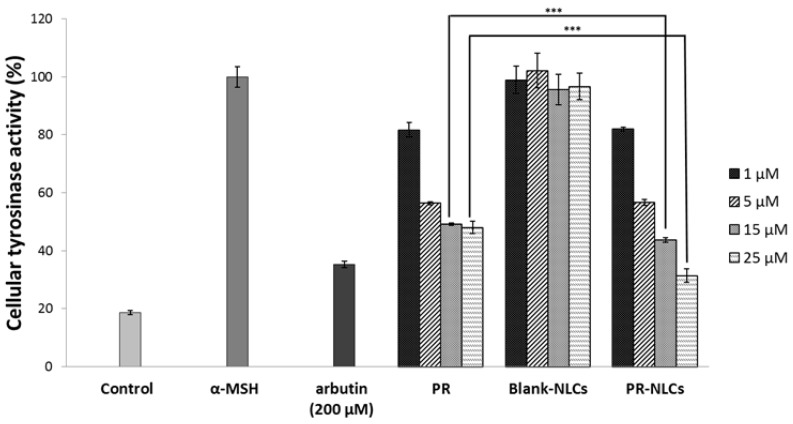
Inhibitory effects of PR, blank-NLCs, and PR-NLCs on cellular tyrosinase activity. *** *p* < 0.001.

**Figure 13:**
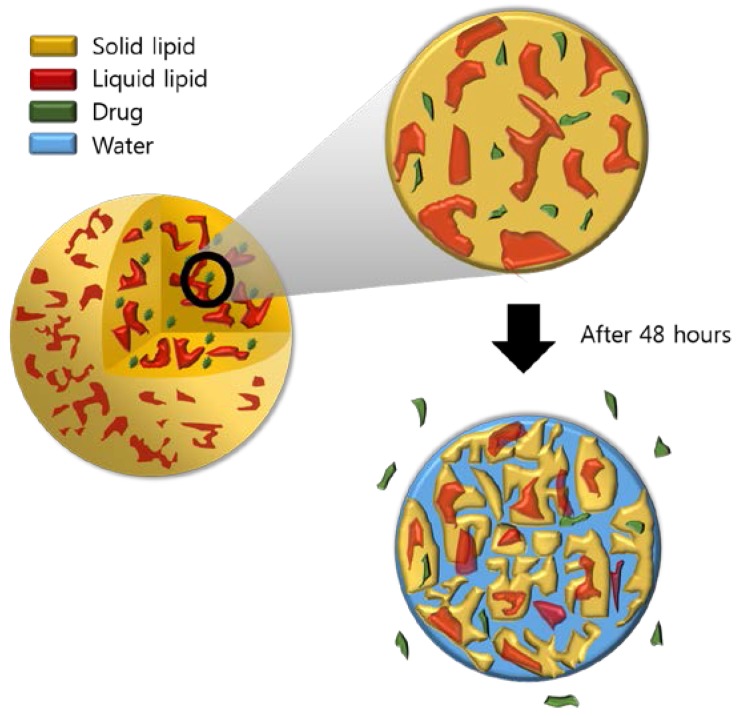
NLC model showing the release of PR.

To evaluate the whitening effect of the PR, blank-NLCs, and PR-NLCs, B16F10 melanoma cells were treated with the formulation at a range of concentrations that showed no toxicity on cell viability. The melanoma cells did not exhibit cytotoxicity at concentrations less than 10 μg/mL for the incubation of 24 or 48 h (data not shown). Melanin synthesis was induced by α-melanocyte stimulating hormone (α-MSH) in B16F10 cells. α-MSH is one of the factors that stimulate pigmentation [34]. As shown in Figure 12, the blank-NLCs had no effects on tyrosinase activity, but the PR and PR-NLCs inhibited α-MSH-induced tyrosinase activity more effectively than arbutin, a whitening agent used as a positive control [35]. Interestingly, the PR-NLCs could more significantly inhibit tyrosinase activity in melanoma cells than PR at concentrations above 15 μM. It was found that the cellular tyrosinase activity was related to the cellular uptake study. Based on the above results, NLCs reduced the amount by 15% compared to PR only for skin whitening, suggesting that NLCs can be used as a nanocarrier in order to effectively apply PR. Taken together, we propose the NLC model blended with both solid lipid and liquid lipid in an amorphous type [36]. PR was slowly released from NLC because NLCs have the ability to increase the solubility of PR due to the amorphous state (Figure 13). Therefore, NLCs containing PR may provide promising formulations that may be able to reduce side effects and maximize efficiency when applied to cosmetic and skin-related diseases. 

## 3. Materials and Methods 

### 3.1. Materials

PR was obtained from IN2BIO R&D Center (Suwon, Korea). Arbutin, 3,4-dihydroxy-L-phenylalanine (L-DOPA), dimethyl sulfoxide (DMSO), 3-(4,5-dimethylthoazol-2yl)-2,5-diphenyl-2H-tetrazolium bromide (MTT), Dynasan^®^, ethylenediaminetetraacetic acid (EDTA), FITC, and α-MSH were purchased from Sigma-Aldrich (St. Louis, MO, USA). Behenic acid, myristic acid, palmitic acid, and stearic acid were obtained from Daejung Chemical (Cheongwon, Korea). Dulbecco’s modified Eagle’s medium (DMEM), fetal bovine serum (FBS), and penicillin–streptomycin were purchased from Gibco (New York, NY, USA). Compritol^®^ 888 ATO and Precirol^®^ ATO 5 were provided by Gattefossé (Saint Priest, Cedex, France). GMS, Labrasol^®^, Miglyol^®^ 812, mineral oil, oleic acid, olive oil, polyvinyl alcohol (PVA), and Tween^®^ 80 were purchased from Samchun Chemical (Pyungtaek, Korea). Lecithin was provided by Junsei Chemical (Tokyo, Japan). D(+)-trehalose dehydrate was purchased from Acros Organics (Morris Plains, NJ, USA). Rhodamine phalloidin and 4’, 6-diamidino-2-phenylindole (DAPI) were purchased from Sigma-Aldrich. High-performance liquid chromatography (HPLC)-grade methanol was obtained from JT Baker (Phillipsburg, NJ, USA). All other chemicals were commercial products of analytical or reagent grade and used without further purification.

### 3.2. Cell Cultures

B16F10 and HaCaT cells were obtained from Korean Cell Line Bank (Seoul, Korea). HaCaT cells and B16F10 cells were cultured with DMEM supplemented with 10% FBS, 100 units/mL of penicillin, and 100 μg/mL of streptomycin in a humidified atmosphere of 5% CO_2_ at 37 °C.

### 3.3. Screening of Solid and Liquid Lipids

The partitioning of PR was measured in Dynasan^®^ 118, behenic acid, palmitic acid, stearic acid, myristic acid, GMS, Precirol^®^ ATO 5, or Compritol^®^ 888 ATO as solid lipids. Briefly, 10 mg of PR were dispersed in a mixture of melted lipid (1 g) and 5 mL of hot distilled water, and then shaken for 10 min in a hot water bath. After cooling, the aqueous phase was separated after cooling and centrifugation at 21,000 × *g* for 15 min [37,38,39]. The drug content in the supernatant was analyzed by HPLC (Agilent 1100 HPLC system). The partition coefficient was calculated as follows:

$$\text{Partition coefficient}=\log(\frac{\text{initial amount of PR}-\text{the amount of PR in aqueous phase}}{\text{the amount of PR in aqueous phase}}).$$

For solubility of PR in mineral oil, Labrasol^®^, oleic acid, Miglyol^®^ 812, or olive oil as liquid lipids, excess PR was dispersed in a microcentrifuge tube containing 1 mL of liquid lipid. The samples were put on an end-to-end lab quake rotator at 8 rpm at room temperature for 72 h in order to achieve equilibrium. The samples were then centrifuged at 13,500 × *g* for 20 min. The supernatant was diluted with methanol and analyzed by HPLC [14]. Each determination was carried out in triplicate.

### 3.4. Preparation of PR-NLCs

The PR-NLCs were prepared by the hot-melted ultrasonic method. GMS as the solid lipid and olive oil as the liquid lipid were mixed at a ratio of 7:3 to form a lipid phase. The aqueous phase was composed of 0.5% (*w*/*v*) PVA, 0.7% (*w/v*) lecithin, and 1.5% (*w/v*) Tween^®^ 80 in 10 mL distilled water. After the two phases were separately heated to 75 °C, the aqueous phase was added to the lipid phase containing PR, and the mixture was homogenized at 15,000 rpm for 5 min to obtain the coarse emulsion. Then, this emulsion was sonicated with a probe-type sonicator for 10 min and quickly cooled in an ice bath to form PR-NLCs. Blank-NLCs was prepared without PR with the same method. Freeze-dried PR-NLCs were prepared using a trehalose by a freeze-dryer FD-1000 (EYELA, Tokyo Rikakikai, Tokyo, Japan).

The PR–lipid melt was prepared without an aqueous phase. GMS and olive oil were heated to above the melting point of GMS to form a lipid phase. PR was dispersed in the melted lipid phase and quickly cooled in an ice bath to form a PR-lipid melt. The lipid–melt was prepared without PR with the same method.

### 3.5. Measurements of Particle Size and PDI

The particle size and PDI were determined by DLS using a Zetasizer Nano S90 (Malvern Instruments, Malvern, UK). Prior to measurement, all the samples were diluted with distilled water to gain an appropriate scattering intensity, and measurements were performed at 25 °C. Each formulation was measured in triplicate, and the results are presented as mean ± SD.

### 3.6. Determination of EE and LC

The EE and LC of PR-NLCs were evaluated using an ultrafiltration method. The PR-NLCs (0.5 mL) were then placed in the upper chamber of a centrifuge tube matched with an ultrafilter device (molecular weight cutoff (MWCO) 100 kDa, Amicon Ultra, Millipore, Billerica, MA, USA) and the centrifuge tube was centrifuged at 14,000 × *g* for 30 min. The aqueous dispersion medium containing the unloaded PR penetrated through the filter membrane into the sample recovery chamber and was detected by HPLC. The EE and LC could be calculated by the following equations:

$$\mathrm{EE}\;\left(\%\right)=\frac{W_{T}-W_{F}}{W_{T}}\times 100$$

$$\mathrm{LC}\;\left(\%\right)=\frac{W_{T}-W_{F}}{W_{L}+W_{T}-W_{F}}\times 100$$

where *W*_T_ is the weight of total amount of PR, *W*_F_ is the weight of unloaded PR in PR-NLCs, and *W*_L_ is the total weight of lipid.

To check the potential accumulation of PR in the ultrafiltration membrane, 0.5 mL of PR solution (1 mg/mL, the solvent was the mixture of methanol:water (7:3)) was added to an ultrafiltration device and the filtrate was withdrawn after centrifuging at 14,000 × *g* for 30 min. HPLC was applied to determine the concentration of samples before and after permeating through the ultrafiltration membrane [15]. The permeation percentage was calculated by the following equation:

$$\text{Permeation percentage}=\frac{C_{\mathrm{after}}}{C_{\mathrm{before}}}\times 100$$

where *C*_after_ is the concentration of PR solution after permeating through the ultrafiltration membrane and *C*_before_ is the concentration of PR solution before permeating the ultrafiltration membrane.

### 3.7. HPLC Analysis

HPLC analysis of PR was performed with the Agilent 1100 HPLC system (Agilent Technology, Santa Clara, CA, USA) equipped ultraviolet (UV) detector [3]. The analytical column was a ProntoSIL 120-5-C18 SH (150 mm × 4.6 mm, 5 µm; Bischoff Chromatography, Leonberg, Germany). The mobile phase composed of methanol and water (70:30, *v/v*) was filtered using a 0.45-µm filter, degassed, and delivered at a flow rate of 0.7 mL/min. The column temperature was maintained at 35 °C and the injection volume was 10 µL. PR was detected at 254 nm.

### 3.8. TEM Analysis

The surface morphology of the PR-NLCs was evaluated by TEM (JEM-1400, JEOL, Tokyo, Japan). The PR-NLCs were diluted with distilled water to observe appropriate images. A drop of sample was placed on a formvar/carbon-coated copper grid (Ted Pella, Redding, CA, USA) and allowed to evaporate under ambient conditions. Then, the samples were negatively stained with 2% (*w/w*) uranyl acetate and air-dried at room temperature for 2 min. The excessive fluid was removed using filter paper. The samples were observed with a JEM-1400 operating at 120 kV and the data were analyzed with the JEOL Simple Measure program (JEOL, Tokyo, Japan).

### 3.9. FT-IR Analysis

In order to assess the interactions between PR and compositions of NLCs, FT-IR analysis was performed on PR only, lipid melt, PR–lipid melt, freeze-dried blank-NLCs, and freeze-dried PR-NLCs. To prepare lipid melt, GMS and olive oil were blended under stirring at 75 °C and then cooled in an ice bath. In the case of PR–lipid melt, it was prepared in the same method with PR. The spectra were recorded using a Thermo Scientific Nicolet 380 Spectrophotometer (Thermo Fisher Scientific, Waltham, MA, USA). A total of 64 scans were used and data were recorded over the range 4000–400 cm^–1^ at room temperature. Background scanning and correction were performed before each measurement.

### 3.10. DSC Analysis

To determine the changes of thermal characteristics for the PR and lipid matrix, DSC analysis was carried out in a Mettler Toledo DSC 1 (Mettler Toledo, Schwerzenbach, Switzerland). The melting enthalpy, onset and melting temperature were analyzed with STAR Software (Mettler Toledo, Schwerzenbach, Switzerland). Accurately weighed samples were placed in an aluminum pan and sealed with a lid. In the scanning process, a heating rate of 10 °C/min was applied in the temperature range from 20 to 180 °C under a nitrogen gas. An empty pan was used as a reference.

### 3.11. XRD Analysis

XRD was studied to determine the crystallinity of the lipid and PR dispersed in the lipid matrix. XRD patterns were recorded at room temperature using a D/Max-2200 Ultima/PC (Rigaku, Tokyo, Japan) with Ni-filtered Cu–Kα radiation powered at 40 kV and 40 mA. The samples were scanned over a 2θ range from 5° to 70° in continuous scan mode using a step size of 0.02°/s.

### 3.12. Stability Test

The stability test was conducted to evaluate the long-term stability of freeze-dried PR-NLCs. The freeze-dried samples were stored at 4 °C in the dark and 25 °C in the daylight. The changes of the particle size, PDI, and EE were determined for a three-month storage.

### 3.13. In Vitro Release Study

An in vitro release study of PR from PR-NLCs was performed in distilled water or 1% sodium dodecyl sulfate (SDS) solution using the dialysis bag method. The dialysis bags were soaked in water for 12 h before use. An amount of 10 mg of PR was loaded in a dialysis bag (Spectra/Por^®^ Cellulose Ester Membrane, MWCO: 25 kDa, Spectrum Labs, Rancho Dominguez, CA, USA) and stirred at 200 rpm at room temperature. The PR dissolved in methanol and 1.5% Tween^®^ 80 were used as the controls. At pre-determined time intervals (1, 2, 4, 8, 12, 24, 36, 48, 60, and 72 h), 1 mL of sample was taken and 1 mL of fresh medium was added to maintain sink conditions.

### 3.14. Cytotoxicity Study

HaCaT cells were seeded in 96-well plates at a density of 5.0 × 10^4^ cells per well in 100 μL DMEM and incubated for 24 h to promote adhesion. Then, the cells were added to different formulations of PR solution, blank-NLCs, and PR-NLCs at a PR concentration ranging from 6.25 μg/mL to 100 μg/mL. The cytotoxicity of blank-NLCs was evaluated with a lipid concentration equivalent to that of PR-NLCs. PR was dissolved in DMSO and then diluted 300-fold with DMEM to obtain a final concentration of 100 μg/mL. Simultaneously, blank-NLCs or PR-NLCs were directly added and diluted with DMEM. After 24, 48, and 72 h, the medium was replaced with 100 μL of MTT solution (5 mg/mL), and cells were incubated at 37 °C for 3 h. Formazan crystals were dissolved in 100 μL of DMSO. Then, the plates were placed in an incubator for 30 min. Finally, the absorbance of each well was measured using a microplate reader (Sunrise, Tecan, Salzburg, Austria) at 570 nm. The cell viability was calculated using the following equation:

$$\text{Cell viability }\left(\%\right)=\;\frac{{OD}_{\mathrm{treat}}}{{OD}_{\mathrm{control}}}\times 100$$

where *OD*_treat_ is the optical density of the treated sample and *OD*_control_ is the optical density of the non-treated sample.

### 3.15. Cellular Uptake Study

The NLCs were labeled with FITC instead of PR, and FITC-NLCs were prepared by the same method of PR-NLCs. In order to determine the cellular uptake of FITC from FITC-NLCs, flow cytometry was used. B16F10 mouse melanoma cells were seeded in a 60-mm dish at a density of 5 × 10^5^ cells per dish in 3 mL growth medium and incubated for 24 h to promote adhesion. Then, cells were treated with a FITC solution or FITC-NLCs (the equivalent concentration of 12.5 µg/mL) at 37 °C for 4 and 8 h. Cells were harvested and centrifuged to remove the supernatant. Subsequently, cells were washed with cold phosphate buffered saline (PBS), fixed with 4% paraformaldehyde in PBS for 30 min, and resuspended in 0.5 mL of PBS. Suspensions were measured by flow cytometry (BD FACS Canto™ II Flow Cytometry, BD Biosciences, Franklin Lakes, NJ, USA). Data analysis was performed using FlowJo software (Tree Star, Ashland, OR, USA).

Confocal laser scanning microscopy was also performed to qualitatively evaluate the cellular uptake of FITC from FITC-NLCs. B16F10 cells were treated with FITC solution or FITC-NLCs (the equivalent concentration of 12.5 µg/mL) at 37 °C for 4 and 8 h. Cells were washed twice with cold PBS, fixed with 4% paraformaldehyde in PBS for 10 min, and then permeabilized with a 1% Triton^®^ X-100 for 5 min. For fluorescence staining, filamentous actin was labeled with rhodamine phalloidin solution at room temperature for 30 min. After washing, cells were incubated with a DAPI solution for 30 min to label nuclei. All the stained samples were washed with PBS prior to observation under a confocal laser scanning microscope (Carl Zeiss Meditec AG, Jena, Germany) equipped with LSM 5 image browser software (Carl Zeiss Meditec AG, Jena, Germany).

### 3.16. Cellular Tyrosinase Inhibition Assay

To determine the whitening effect, cellular tyrosinase assay was performed in B16F10 melanoma cells. Cells were seeded in a six-well plate at a density of 2 × 10^4^ cells per well. Then, cells were exposed to α-MSH (100 nM) for one day and treated with a sample including various concentrations of PR or PR-NLCs and α-MSH. After two days, cells were washed with cold PBS and lysed with 0.1 M sodium phosphate buffer (pH 6.8) containing 5 mM EDTA and 1% Triton X-100 in ice box for 30 min. After centrifugation of the lysate at 21,000 × *g* at 4 °C for 30 min, cellular tyrosinase activity was measured in the resulting supernatant. The protein level in the supernatant was determined by bicinchoninic acid assay. The cellular tyrosinase activity was then measured in a 96-well plate at 150 µL of the reaction mixture contained 0.1 M sodium phosphate buffer (pH 6.8), 0.1% L-DOPA, and supernatant equivalent to 40 µg of protein. After incubation at 37 °C for 1 h, tyrosinase activity was quantified by measuring the absorbance at 475 nm. The cellular tyrosinase activity was calculated using the following equation:

$$\text{Cellular tyrosinase activity }\left(\%\right)=\;\frac{{OD}_{\mathrm{treat}}}{{OD}_{\mathrm{control}}}\times 100$$

where *OD*_treat_ is the optical density of the treated sample and *OD*_control_ is the optical density of the control sample.

### 3.17. Statistical Analysis

In this paper, all data are reported as the mean ± SD. Student’s *t*-test was used to compare two different groups of samples. The acceptable level of significance was established at *p* < 0.001.

## 4. Conclusions

In this study, PR-NLCs were successfully prepared through the screening of solid and liquid lipids for PR by the hot-melted ultrasonic method. PR-NLCs have a small particle size of 57.9 ± 1.3 nm, narrow size distribution of 0.24 ± 0.01, high EE of 93.1 ± 4.2%, spherical morphology, and sustained release of 48% in 72 h. Also, the incorporation of PR into NLCs could lend excellent physicochemical stability and photostability at 4 °C in the dark and 25 °C in the daylight during three-month storage. A cytotoxicity study on HaCaT cells indicated that PR incorporated in the lipid matrix minimizes the cytotoxic effect. A cellular uptake study and tyrosinase inhibition assay on B16F10 cells indicated that NLCs could efficiently deliver PR into melanoma cells. This manuscript is the first work to show the efficacy of PR-NLCs with a cellular tyrosinase inhibition assay.

## Figures and Tables

**Table 1:** The particle size, PDI, EE, and LC of blank-NLCs and PR-NLCs (*n* = 3, mean ± SD).

Formulation	Particle Size (nm)	PDI	EE (%)	LC (%)
Blank-NLCs	55.6 ± 1.2	0.25 ± 0.01	-	-
PR-NLCs	57.9 ± 1.3	0.24 ± 0.01	93.1 ± 4.2	8.5 ± 0.4
